# Activity Space and Functional Outcomes in Frail Older Persons Using GPS analysis

**DOI:** 10.1093/geroni/igab046.1989

**Published:** 2021-12-17

**Authors:** Sandra Lau, Frerk Mueller- von Aschwege, Tania Zieschang, Juergen Bauer, Andreas Hein, Rebecca Diekmann

**Affiliations:** 1 Carl von Ossietzky University Oldenburg, Oldenburg, Niedersachsen, Germany; 2 OFFIS e.V. - Institute for Information Technology, Oldenburg, Niedersachsen, Germany; 3 Heidelberg University, Heidelberg, Baden-Wurttemberg, Germany

## Abstract

With increasing age, walking becomes a main functional ability to participate in activities of daily living and supports independence and mobility. Frailty in older, multimorbid patients has a negative impact on physical activity and may reduce the personal activity space (AS). In this pilot study, GPS data were used to identify walking tracks to define individual AS and to compare functional performance in frail older persons. GPS data of 20 community-dwelling adults (84.5(±5.2)years, 85% women, mean frailty phenotype 1.9 (70% ≥2)points) were analyzed using a customized software to assess individual AS over a ten-months period. A geriatric home assessment including Short Physical Performance Battery (SPPB), gait speed (GS) and Timed-up-and-Go (TUG) was conducted monthly. GPS analysis revealed three different walking types presenting AS similarities: Type A walkers prefer smaller short walks nearby the home while Type B can be characterized by taking larger regular walks. Type C presents the widest AS using different transportation modes, but only a moderate number of walks. Mean group difference in functional performance of Type A walkers showed significantly reduced GS (0.45(±0.1)m/s), TUG (23.4s(±4.9)) and SPPB scores (3.8(±0.8) points; p<0.05) compared to Type C (0.82(±0.1)m/s (GS); 13.2(±1.4)s (TUG); 7.0(±1.3) points (SPPB)). Functional performance of Type B walkers (0.63(±0.2)m/s (GS); 17.1(±4.4)s (TUG); 6.5(±2.4)points (SPPB)) revealed significantly higher SPPB scores compared to Type A (p<0.05). Walks and individual AS can be mapped via GPS under everyday conditions. High heterogeneity within frail older people was observed. Persons with lower functional performance showed a reduced AS and physical activity.

